# Critical Design and Characterization Methodology for a Homemade Three-Axis Fluxgate Magnetometer Measuring Ultra-Low Magnetic Fields

**DOI:** 10.3390/s25133971

**Published:** 2025-06-26

**Authors:** Hava Can, Fatma Nur Çelik Kutlu, Peter Svec, Ivan Skorvanek, Hüseyin Sözeri, Çetin Doğan, Uğur Topal

**Affiliations:** 1 TÜBİTAK National Metrology Institute (UME), 41470 Kocaeli, Turkey; hava.can@tubitak.gov.tr (H.C.); fncelik2018@gtu.edu.tr (F.N.Ç.K.); huseyin.sozeri@tubitak.gov.tr (H.S.); cetin.dogan@tubitak.gov.tr (Ç.D.); 2 Department of Electronic Engineering, Gebze Technical University, 41400 Gebze, Turkey; 3 Institute of Physics, Slovak Academy of Sciences, 84511 Bratislava, Slovakia; peter.svec@savba.sk; 4 Institute of Experimental Physics, Slovak Academy of Sciences, 04001 Košice, Slovakia; skorvi@saske.sk

**Keywords:** fluxgate magnetometer, ultra-low-field magnetic sensors, soft magnetic alloys, measurement techniques, calibration

## Abstract

This paper presents the design, fabrication, calibration, and comprehensive characterization of a homemade tri-axial fluxgate magnetometer. The magnetometer, utilizing a ring core configuration, was developed to measure ultra-low magnetic fields with high sensitivity and stability. Critical stages from material selection to sensor geometry optimization are discussed in detail. A series of critical characterization processes were conducted, including zero-field voltage determination, scale factor calculation, resolution measurement, noise analysis, bias assessment, cross-field effect evaluation, temperature dependency, and bandwidth determination. The sensor demonstrated a minimum detectable magnetic field resolution of 2.2 nT with a noise level of 1.1 nT/√Hz at 1 Hz. Temperature dependency tests revealed minimal impact on sensor output with a maximum shift of 120 nT in the range of 60 °C, which was effectively compensated through calibration to less than 5 nT. Additionally, the paper introduces a model function in matrix form to relate the magnetometer’s output voltage to the measured magnetic field, incorporating temperature dependency and cross-field effects. This work highlights the importance of meticulous calibration and optimization in developing fluxgate magnetometers suitable for various applications, from space exploration to biomedical diagnostics.

## 1. Introduction

Advances in science and technology directly contribute to the development of new, highly sensitive, and more accurate measuring devices. Among these, sensor systems designed for magnetic field measurement have a particularly important role. Magnetic sensors find applications in many different fields such as the space sector, archaeology, medicine (imaging organisms), mineral exploration, and defense (mine and submarine detection, navigation, etc.) [1,2,3,4]. Common types of low-field sensors include SQUID (Superconducting Quantum Interference Device), search coils, Hall effect, proton precession, optical pumping, and fluxgate magnetometers [5,6,7]. In these sensors, magnetic fields from 10^−3^ T down to 10^−10^ T can be measured directly with fluxgate magnetometers, which can operate in a wide temperature range in a robust way with a good electronic and sensor design [8,9]. Considering the diversity and scope of the application area, fluxgate magnetometers are the most widely used low-field magnetic field sensors. In addition to high sensitivity and measurement accuracy, the ability to directly measure the vectoral components of a magnetic field and the ability to measure very low magnetic field strength within electromagnetic noise and a wide temperature range make these sensors advantageous compared to others [10]. With recent advances in material science, the internal noise of these sensors has now been reduced to just a few tens of pT/√Hz, nearly 100 times lower than it was a decade ago. They have the ability to measure the magnitude of magnetic field strength from DC to several hundreds of Hertz [11]. Therefore, with today’s technology, it is possible to examine magnetic rocks having very small residual magnetization (paleomagnetic examination), to find the direction of air vehicles as a part of navigation systems and to monitor the magnetic fields of biological systems such as the heart for diagnostic purposes using fluxgate sensors.

The magnetization characteristic of the ferromagnetic cores that are a part of fluxgate sensors is the main factor determining its performance [12]. In most of the fluxgate sensors in the literature, the harmonics of the excitation signal induced in the sensing coil as a result of the co-application of AC and DC fields are processed as sensor signals. Among these, the second harmonic one is evaluated to be the signal of the sensor due to its high intensity and perfect linear variation with the applied DC field [13,14]. A higher degree of nonlinearity in the magnetization curves (M-H or B-H) of the core material results in increased 2f signal intensity. Additionally, using ferromagnetic cores with low magnetic hysteresis minimizes sensor heating and, consequently, reduces power consumption. The structural homogeneity of the cores also appears to be an important parameter affecting the performance of the sensor. Local structural defects such as production-related impurity atoms, microstresses, and atomic dislocation of the core negatively affect the coordinated domain wall movement, which determines the magnetization characteristics of the cores, and increase the sensor noise level [3,8,15,16]. The saturation magnetostriction coefficient (λs), known as an indicator of shape and size changes during the magnetization of ferromagnetic materials, is also a key factor influencing the noise level in fluxgate sensors. Materials exhibiting soft magnetic behavior, used as the core of fluxgates, can generally be classified into three groups: iron–silicon alloys, amorphous/nanocrystalline alloys, and nickel–iron alloys (permalloy). Iron–silicon alloys, generally used as transformer cores, are known as electrical steel in the literature. While their high Curie temperature (≥500 °C) may help to reduce the temperature dependence of fluxgate sensors, the high magnetic saturation field could negatively impact sensor sensitivity and increase noise levels. Nickel–iron alloys, commonly known as permalloy, can be used across a broad range of nickel compositions, from 30% to 80% by weight. A high Ni amount provides low magnetic permeability and causes low electrical resistance. Special Ni-Fe ratios can achieve near-zero magnetostriction and a magnetic permeability of 300,000. At 80% nickel by weight, the coercive field of these alloys drops to 0.004 A/cm, with a saturation magnetization around 0.7 T. These materials, with a Curie temperature of approximately 360 °C, were used as sensing cores when fluxgate magnetometers were first developed. However, the noise level remained in the range of a few hundred pT/√Hz at 1 Hz. Cobalt-based alloys with an amorphous structure and a thickness of approximately 20 µm can be produced through the rapid-quenching melt-spinning technique. These alloys, with exceptionally high magnetic permeability, are well suited for fluxgate applications because of their low coercivity and saturation magnetization field. The typical saturation magnetization value of around 0.6 T, the Curie temperature of around 200 °C, and electrical resistivity of 1.4 µΩm makes these alloys usable in a wide temperature range as a detection core of fluxgate sensors.

Studies on fluxgate sensors mainly focus on three basic geometries: rod-type, racetrack, and ring core [17,18,19]. Among these geometries, rod-type ones are known to have the worst sensitivity and noise levels. The reason is the open-ended magnetic flux loop. Magnetic flux lines bend at the endpoints of the rod core, which negatively contribute to the noise level caused by the magnetic component. On the other hand, there may be cases where this geometry is advantageous compared to others. For example, these types of sensors are less sensitive to magnetic field components outside the detection direction compared to other geometries. This reduced sensitivity, known as the cross-field effect, is due to the sensor’s high demagnetization coefficient in directions other than the detection axis. Rod-type geometry is the most suitable geometry for application areas that require local magnetic field information like obtaining magnetic information on credit cards. The ring core geometry has been the most preferred fluxgate geometry due to its advantages in controlling the cross-field effect, zero offset value, wide dynamic measurement range, stability, high sensitivity, and resistance to external environmental conditions (robustness). For instance, if increased measurement sensitivity is desired and the upper limit is not a concern, then the ring diameter should be enlarged. Conversely, if the upper limit is important, then the diameter can be reduced. In short, the ring diameter can be determined according to the desired measurement range. In conclusion, as evidenced by the scientific literature on fluxgate sensors, many critical studies need to be carried out with special heat treatments, from determining and optimizing the sensor geometry to adapting it to the electronic circuit, etc.

To ensure the accuracy of equipment, tools, or devices, calibration must be performed periodically under specific critical conditions to increase efficiency, optimize resources, and guarantee the suitability together with compatibility of products and services. For example, if the magnetometers used for navigation purposes in aircraft are not calibrated correctly, then there may be serious consequences that may lead to the loss of the vehicle. Ensuring measurement accuracy is especially critical for detecting ultra-low signal magnitudes generated by physical quantities. These signals easily interfere with the environmental signal sources like electromagnetic signals generated by other devices and harmonics in the main line. When we evaluate the calibration of fluxgate sensors, there are also important issues to consider. For instance, magnetic field components created by the sources located in the measurement area (such as the Earth) can be well above the nT level, which is too high to correctly calibrate the fluxgate sensor. In order to ensure the correct formulation between the sensor output voltage and the measured magnetic field magnitude, internal factors such as sensor geometry and dependence on environmental temperature must be correctly determined and evaluated.

In this paper, we will present the critical stages of design and fabrication processes of a homemade three-axis ring core fluxgate magnetometer together with its calibration and characterization in full dynamical range. Although the sensitivity of the developed magnetometer is relatively worse than the available and used sensors, its robustness to environmental conditions such as temperature and vacuum is quite good compared to its competitors. All critical points identified in the characterization experiments (such as temperature dependency, cross-field effect, supply voltage dependency, and read-out frequency) will be discussed. A model function relating the output signal to the measured magnetic field, which includes parameters such as the zero offset value including its temperature dependency, the scale factor with its temperature dependency, and the cross-field effect will be developed in a matrix form. To our knowledge, there is a clear lack of metrological articles in the scientific literature that address the challenges encountered in characterizing ultra-low-field sensing magnetometers. Many of the techniques discussed in this text will also be useful for characterizing other low-field sensors, from atomic-based sensors to Hall effect sensors.

## 2. Analysis and Discussion

### 2.1. Critical Points on the Design of Fluxgate Sensors and the Electronic Circuit

If we place a magnetic core with a coil wrapped around it under any variable magnetic field, that is, if there is any magnetic flux change along the coil system, then a voltage is induced in the coil in accordance with Faraday’s induction law. This induced voltage is expressed with the time variation of the magnetic flux:

(1) $$\;Vi=\frac{d\phi }{dt}=\frac{d\left(NA\mu_{0}\mu_{r}\left(H\right)H\left(t\right)\right)}{dt}$$

Here, N is the number of turns, A is the cross-sectional area, µ_0_ is the magnetic permeability of the space, µ_r_(H) is the relative magnetic permeability of the core, and H (t) is the total magnetic field applied to the coil. If this equation is written term by term, then Equation (2) is obtained.

(2) $$Vi=NA\mu_{0}\mu_{r}\left(H\right)\frac{dH\left(t\right)}{dt}+N\mu_{0}\mu_{r}\left(H\right)H\left(t\right)\frac{dA\left(t\right)}{dt}+NA\mu_{0}H\left(t\right)\frac{d\mu_{r}\left(H\right)}{dt}.$$

**Figure 1:**
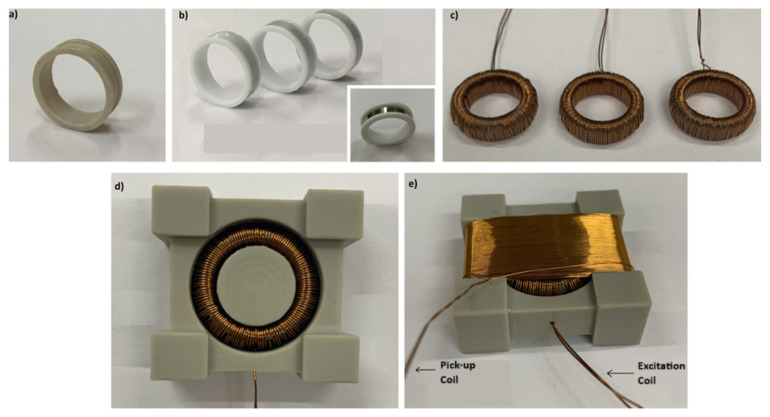
(**a**) The ring template made of GF30 (a 30% glass-fiber-reinforced polyether ether ketone). (**b**) The ribbon wrapped around the GF30 template. (**c**) Toroidal excitation coil. (**d**) View of the excitation coil placed in the pick-up template. (**e**) Latest state of the sensor for the measurement of the one-axis component of the ambient magnetic field.

The third term occurs due to the fluxgate effect as a result of the time-dependent magnetic permeability of the core [17,19]. For the formation of time-dependent magnetic permeability, we need an AC field of H_AC_ = h_0_∙sin(ωt), which is denoted as a modulating or excitation field in fluxgate magnetometry. The total magnetic field together with the ambient DC field will be H(t) = h_0_∙sin(ωt) + H_DC_. The magnetic core needs to be deeply saturated by the modulation field in order to reduce the magnetic noise in fluxgate sensors. The optimization of the amplitude of the AC signal by changing the amplitude of the AC current driven to the excitation coil is also important in terms of the power consumption of the sensor. In the present work, the design of a ring core fluxgate sensor with a diameter of 15 mm was considered. Initially, six layers of cobalt-based ribbons, each 20 µm thick and 3.0 mm wide, were gently wrapped around a ring template made of GF30 (a 30% glass-fiber-reinforced polyether ether ketone) in a channel matching the ribbon width (Figure 1a,b). It is important that the end points of the ribbon coincide. Otherwise, a bias will be generated at the output voltage of the sensor. The magnetic permeability and the saturation magnetization field of the ribbons were ~10,000 and ~0.6 T, respectively. In order to increase the sensitivity, the permeability of the ribbons can be increased to more than 100,000 by annealing at around 300 °C for 1 h, but in such a case it must be considered that the ribbons will be fragile and vulnerable to mechanical shocks. The number of ribbon layers can be increased to up to 8 or more to increase the signal strength, but in this case the signal noise may also increase. So, this parameter needs to be optimized. There are several reasons to use GF30 as a template material such as its high mechanical strength and rigidity, low moisture absorption, and high thermal stability. This support material provides the minimization of the mechanical stress acting on the ribbon due to the mismatch between the thermal expansion coefficients of the ribbon and the template material, if it is used in environments where temperature variations are high, like space atmosphere. It should be noted that machinable ceramics can also be useful due to their low thermal expansion, but they require careful mechanical turning and are relatively expensive. Another important point to consider in the design is the determination of the ring diameter. If the sensitivity of the sensor is the dominant target, then the diameter must be increased even up to more than 25 mm. In this case, the upper range of the sensor will be smaller than the Earth’s field of 60 μT. If the sensitivity is of secondary importance and its usability in daily life is critical, then the diameter can be smaller like in the present design. The second stage of the sensor design is the construction of the excitation coil. In our case, a toroidal winding was performed around the ring core with a copper wire of 300 μm in diameter (Figure 1c). The insulating layer of copper wire is important for withstanding the high currents and avoiding electrical short circuits. We used space-qualified grade 2 copper wire, which can endure currents up to 250 mA for long periods. In order to magnetize all parts of the ribbons homogeneously and to minimize the final noise level of the sensor, the number of turns at one half of the toroid must be equal and geometrically symmetric with the other half. Similarly, the total number of turns of the toroidal winding is critical for deep saturation of the core. Depending on the power restriction, the number of winding layers can be as high as five, but we made three layers in our sensors, with ~375 total turns. As will be mentioned in the following sections of the text, the amplitude of the current passing through the excitation coil can be adjusted by an appropriate capacitor. The last stage of the sensor construction was the design of the pick-up coil. Again, GF30 was used as the template material of the pick-up coil for the same reasons mentioned before (Figure 1d). The windings were made using space-qualified grade 2 copper wire with a diameter of 200 µm for the measurements of three-axis magnetic field components (Figure 1e). The total number of turns was ~480. We must note that the optimal number of turns may change depending on the ring diameter. After placing the ring into the center of the pick-up coil, it was rotated carefully to minimize the zero offset value, monitoring the signal generated over pick-up coil to be near zero. Otherwise, a bias and a difference between the negative–positive field direction of the magnetic field appears on the output voltage of the sensor.

**Figure 2:**
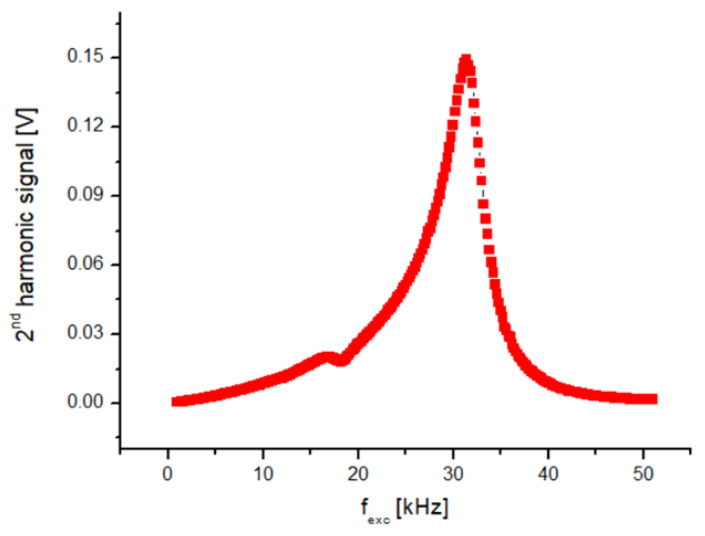
Excitation frequency versus V2f signal strength.

**Figure 3:**
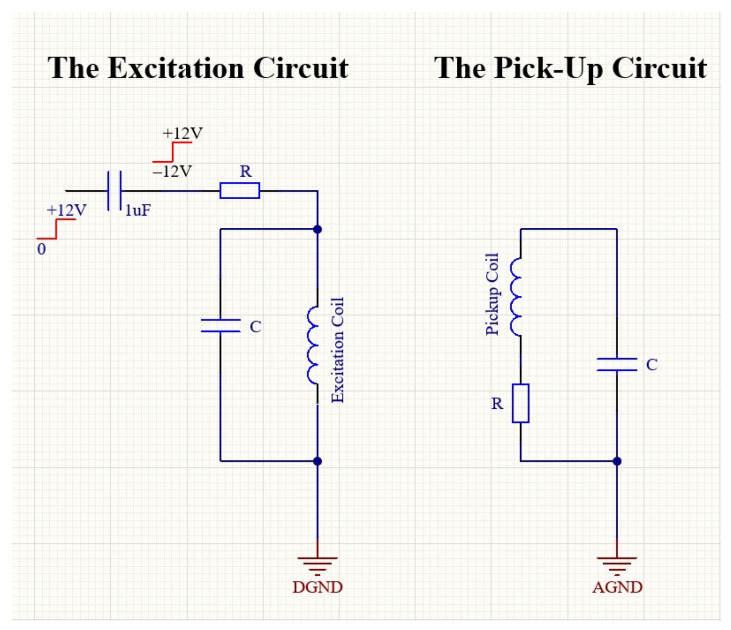
Excitation (**left**) and the pick-up circuit (**right**) of the fluxgate magnetometer.

**Figure 4:**
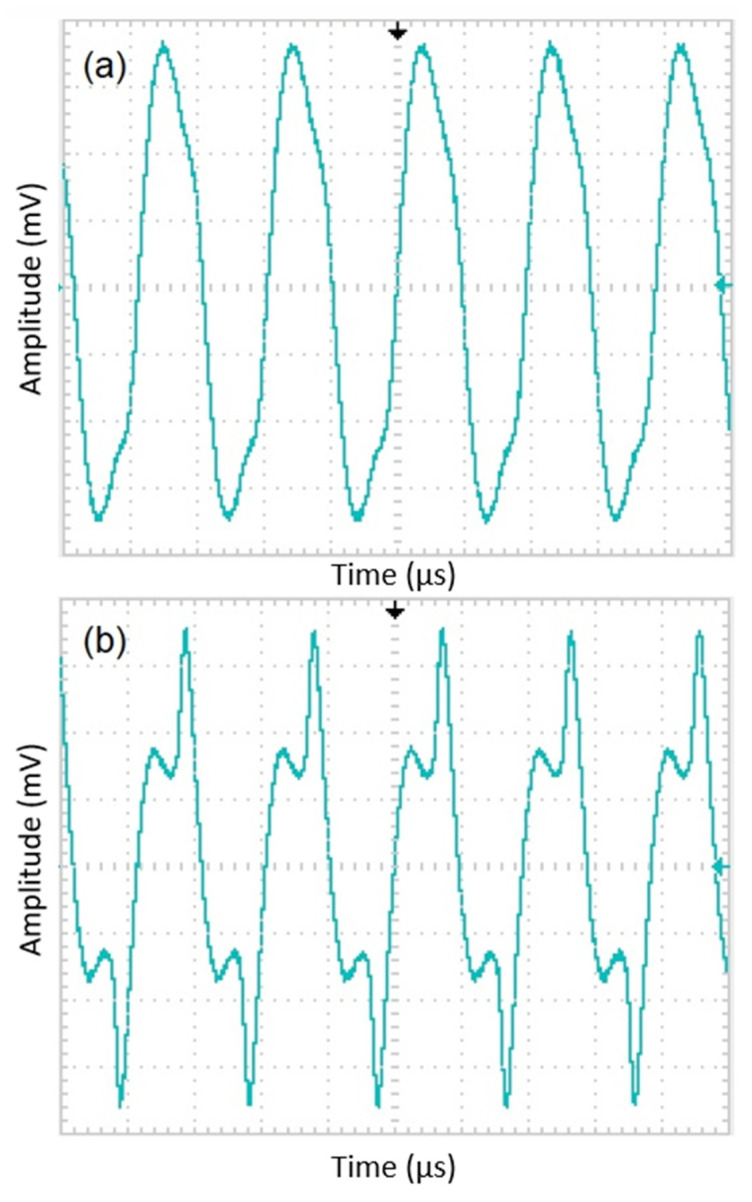
The possible current wave forms passing over the excitation coil with duty cycle of 50%. (**a**) Not suitable; the cores are not saturated. (**b**) Suitable; the cores are deeply saturated.

**Figure 5:**
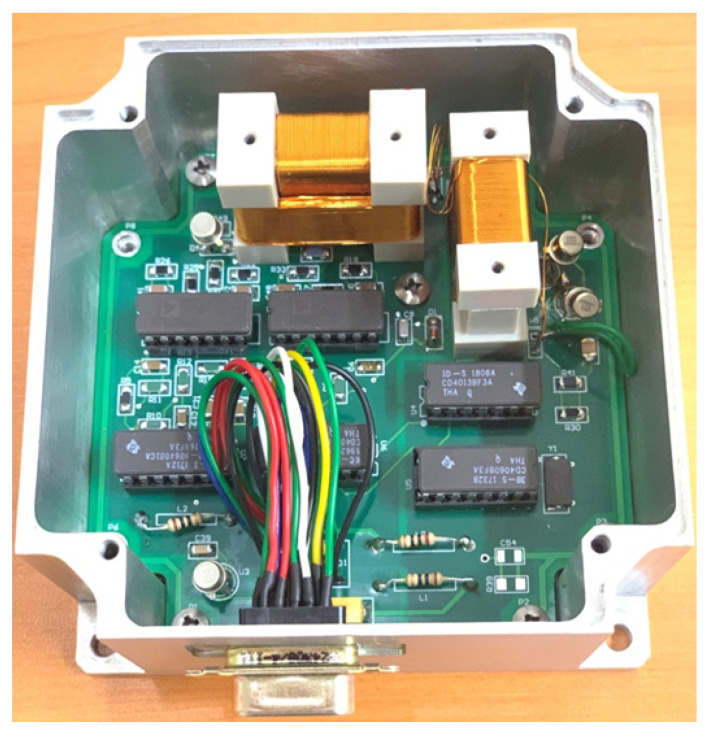
Final version of the homemade three-axis fluxgate magnetometer.

In order to facilitate electronic design and ensure compatibility with the sensor, the first step is to determine the excitation frequency (f) and then amplify the sensor’s output signal at 2f frequency. In order to determine the excitation frequency, a sinusoidal AC current with sufficient amplitude to saturate the cores was passed through the excitation coil with 100 Hz steps from 1 to 50 kHz. Higher frequencies are undesirable due to potential noise caused by eddy current losses on the surface of the ribbons. A constant DC field was additionally applied parallel to the sensing direction of the sensor using a Helmholtz coil to generate the 2f signal in the pick-up coil. Then, the 2f data were collected by a lock-in amplifier. Figure 2 shows the f_exc_–V_2f_ curve of one of the sensors examined in this work. As seen from Figure 2, the V_2f_ curve shows a sharp peak at the excitation frequency of 31.4 kHz. At first glance, it might be thought that this excitation frequency is suitable. However, this self-resonance region, caused by the parasitic capacitance of the coil, should be avoided as much as possible, and the sensor should work far away from the self-resonance frequency [20]. It must be noted that parasitic self-capacitances existing due to windings create a resonant circuit, which is undesirable as the value of the parasitic capacitance may become unstable with increasing time and temperature. When evaluated from an electronic perspective, frequencies that are powers of 2, such as 4, 8, 16, and 32 kHz, are easy to produce using a crystal oscillator. Figure 2 reveals that 32 kHz is quite close to the self-resonance of the excitation coil; therefore, it was abandoned, and 16 kHz was chosen as the excitation frequency. It is important to determine the supply voltage (e.g., 9 V, 12 V, or 15 V) of the designed magnetometer according to factors such as power consumption, cost, and ergonomics. In our design, we preferred 12 V. Now, the initial tests of the excitation circuit, shown in Figure 3 (left), can begin. One half of a square wave (0–12 V) at 16 kHz generated by a function generator is driven to the excitation circuit with a duty cycle of 50%. By means of the 1 μF capacitor, the current form shown in Figure 4 must pass through the tank circuit where the excitation coil is located. As seen from Figure 4, the waveforms of the current through the excitation coils show variation. The quantifying criterion for a suitable current waveform is symmetry breaking on the driven current waveform, which is required for obtaining its harmonics based on the sensor’s output as a result of the field dependence of the magnetic permeability of the core material. If the shape of the current is sinusoidal (Figure 4a), which was measured by a Tektronix P6022 current probe, then it means the ribbons are not saturated magnetically. The current waveform shown in Figure 4b is attempted to be obtained by adjusting the capacitor value in the tank. By changing the value of the resistor at the entrance of the tank, the power consumption of the circuit is adjusted. Finally, the magnitude of the output signal of the sensor is adjusted by changing the value of the capacitor and the resistor in the tank of the pick-up circuit (Figure 3 (right)). In the pick-up circuit, capacitance adjustment is required to increase the intensity of the output signal, while resistor adjustment is required to minimize signal dependency. In this way, stability against temperature or voltage fluctuations is ensured. Now, the sensors (x-, y-, and z-axes) and the circuit are integrated and soldered to the homemade PCB as shown in Figure 5. It is important to note that the three-axis magnetometer analyzed in the present work is not a detection element that senses all three components of the magnetic field at a single point or, at the very least, at approximately one location. Instead, the sensor in question detects individual values at different places in space as shown in Figure 5.

This circuit includes zero-feedback electronics to provide stability and robustness in addition to the excitation and pick-up circuits. If possible, no magnetic electronic or mechanical parts should be used around the sensor. For example, it would be more appropriate to change the integrated circuit elements seen here to SMD components. Notice that even the corners of the PCB inside the box in Figure 5 are held by titanium screws, which are not magnetic. Now, the three-axis fluxgate magnetometer is ready for functional tests as seen in Figure 5. Notice that the sensor on the right side uses one core with a single winding to measure the x-component of the ambient field. On the other hand, although the sensor on the left side also uses one core, it has two windings to measure the y- (inner-winding) and z- (outer-winding) components of the ambient field.

### 2.2. Critical Points on Functional Tests of the Fabricated Fluxgate Sensor

The functional tests began with the determination of the voltage output values of the axes in the zero-field. For this purpose, the magnetometer was placed into a six-layered mu-metal chamber, and the voltage values read from the axes were recorded by using an Agilent34401A digital multimeter (Agilent Technologies Inc, Santa Clara, CA, USA). In fluxgate sensors, the sensor output voltage should be zero at zero magnetic field, negative for magnetic fields antiparallel to the sensor detection direction, and positive for the parallel direction. On the other hand, due to restrictions on the output voltages to 0 V and 5 V for the present work, the design was realized for a special application area, and a voltage output of nearly 2.5 V was expected in the zero field. Similarly, it was expected that the voltage output of 0 to 2.5 V approximately corresponds to the magnetic fields antiparallel to the measurement direction, and 2.5 V to 5 V approximately corresponds to the magnetic fields parallel to the measurement direction. We found the zero-field voltage values to be 2.3759 V, 2.3693 V, and 2.3574 V for x-, y-, and z-axes, respectively. It must be noted that since the mu-metal chamber cannot provide superior magnetic shielding (i.e., a few nT may remain inside), the magnetometer must be placed inside the chamber in different directions, and the average must be taken for the determination of the correct zero-field output. For instance, 2.3690 V, 2.3689 V, and 2.3702 V were measured at different locations of the magnetometer inside the chamber, and its average was taken to be the zero-field value of the y-axis.

#### 2.2.1. Scale Factor

**Figure 6:**
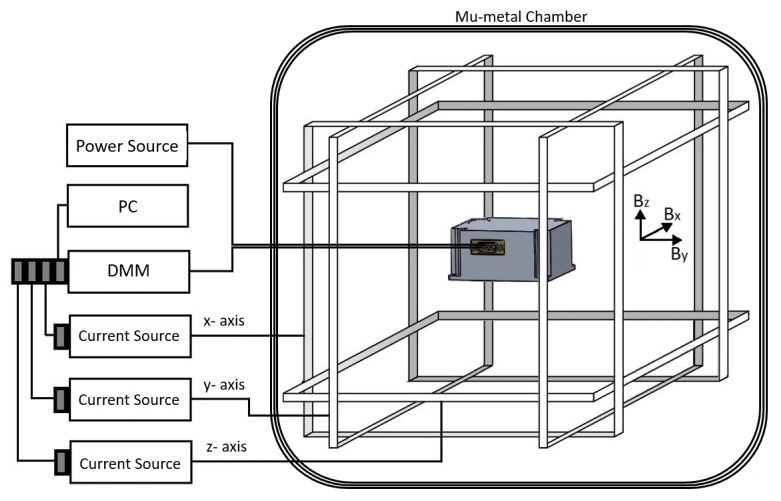
Experimental set-up for the characterization of the magnetometer. Note: mu-metal chamber is included only to demonstrate ideal measuring conditions.

**Figure 7:**
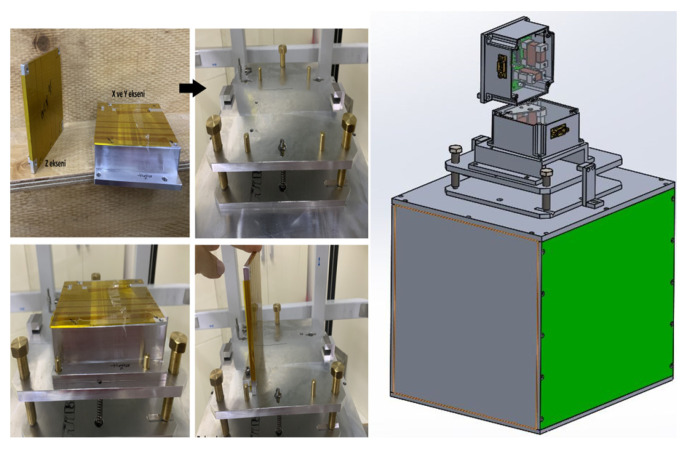
Homemade magnetometer centering fixture.

**Figure 8:**
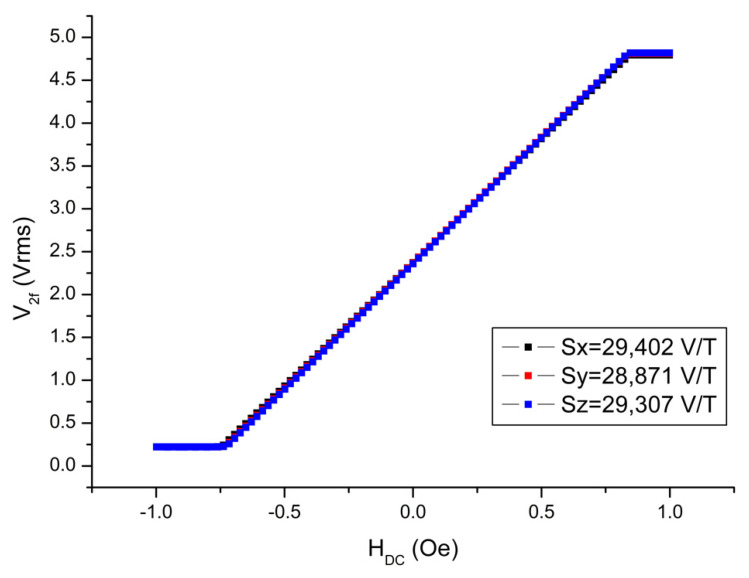
The scale factor measurements of the designed fluxgate magnetometer for x-, y-, and z-axes. Note: 1 Oe = 10^−4^ T.

The next measurement was the determination of the scale factors. For this experiment, the magnetometer was placed into the center of a three-axis calibrated Helmholtz coil having a coil constant of 4.3402 Oe/A with an uncertainty of 0.01% (0.43402 mT/A) as shown in Figure 6, and the output signals read from the magnetometer were recorded with software coded using the LabVIEW (v. 2014 SP1) program for different values of the DC current applied to the coil. Here, it should be ensured that the measurement axis is parallel to the direction of the magnetic field created by the Helmholtz coil, which was supplied with a special homemade fixture consisting of nonmagnetic parts as shown in Figure 7. It is also crucial that environmental magnetic field components, such as the Earth’s field component perpendicular to the measurement axis, are avoided as much as possible. This can be achieved by driving a sufficiently large DC current to the perpendicular axes of the Helmholtz coil and checking the magnitude of the environmental field in that direction using an another traceable magnetometer. However, the most appropriate solution is realizing this experiment, if possible, in a Helmholtz coil placed inside the mu-metal chamber (See Figure 6 for demonstration). In this case, the unwanted environmental magnetic field will already be isolated, but the coil must be re-calibrated due the influence of the shield, which may change the coil constant by some amount. We must note here that the calibration of the Helmholtz coil used in the present work was conducted by a low-field NMR teslameter, which is traceable to universal constants with an uncertainty of 10 ppb. Similarly, current measurements must also have measurement accuracy at least at the nanoampere level. Together with the contribution of the current uncertainty, the total uncertainty of the Helmholtz coil was 0.01%. In order to understand whether there was any hysteresis in our measurements, a cycle was made between − and + maximum current values, and the driven current values were converted into magnetic field units by multiplying them with the constant of the Helmholtz coil (e.g., 0.43402 mT/A). Graphs of the magnetometer output signal to the applied DC field (V_2f_–H_DC_) were drawn, and magnetometer scale factors were calculated from the slopes of the best-fit curves in the linear region. As can be seen in Figure 8, the scale factors for each measurement axis are nearly identical, with values of 29,402 V/T, 28,871 V/T, and 29,307 V/T for the x-, y-, and z-axes, respectively. The scale factors can be increased or decreased by changing the value of resistors connected to the pick-up coils of each axis. In our case, we tried to adjust it to the same value. The R^2^ values, which give information about the quality of fit to the experimental data, were determined to be 0.99992 for all axes of the magnetometer. This means that its linearity is 99.992%. In other words, the nonlinearity of the transducer (from volt to T) is just 0.008%. As can be seen, each axis reaches saturation after approximately 0.2 V at the negative maximum and 4.8 V at the positive maximum. Here, we must note that the restriction on the minimum and maximum voltage values are due to diodes in the electronic circuit required for a special application of the designed magnetometer. This restriction can be eliminated easily for any other applications.

#### 2.2.2. Resolution

**Figure 9:**
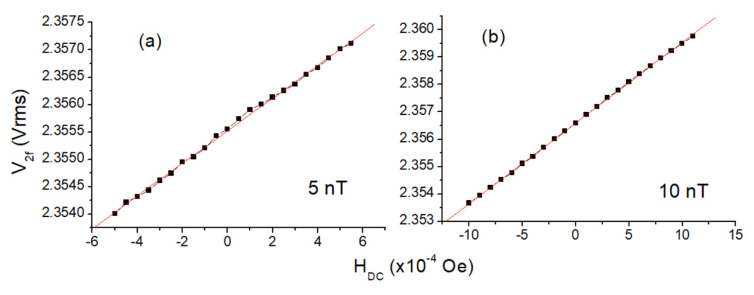
V_2f_–H_DC_ curves of the magnetometer for (**a**) 5 nT and (**b**) 10 nT steps of the ambient field. Note: 1 Oe = 10^−4^ T. Red lines are linear fit lines to calculate the model function of the sensor which is different from the black lines.

In order to determine the minimum detectable magnetic field (resolution), the ambient magnetic field was changed in a narrow range with smaller steps (5 nT and 10 nT; see Figure 9), and the magnetometer output signals were recorded. In these measurements, a Keithley 220 current source with 1 nA sensitivity was used. In resolution measurements, the data collection must be completed as soon as possible in order to minimize the environmental effects; so, the measurement needs to be repeated several times for assurance. If possible, the realization of this experiment in a magnetically shielded medium is also suggested. Otherwise, it will be quite difficult to determine the real resolution of the magnetometer due to environmental magnetic pollution. As can be seen in Figure 9, the designed magnetometer can detect a 5 nT DC field easily.

#### 2.2.3. Noise Analysis

**Figure 10:**
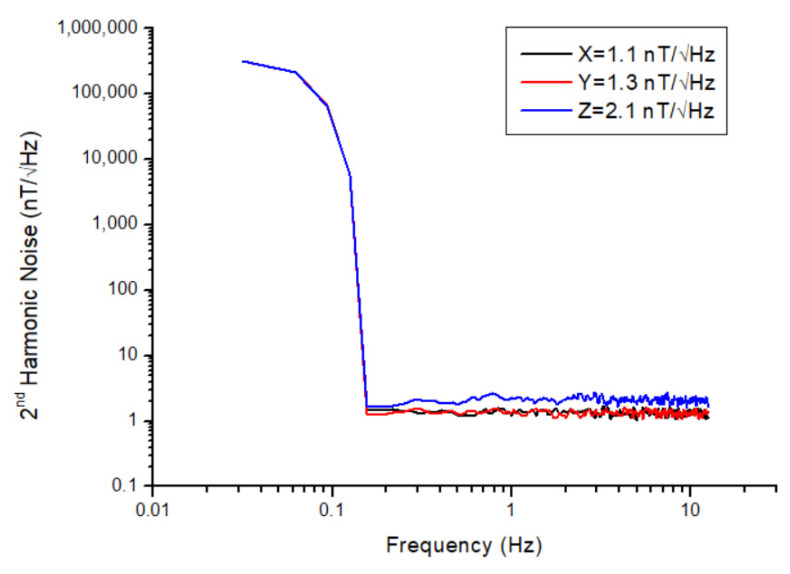
Noise spectrum of the x-, y-, and z-axes of the designed magnetometer.

During noise analysis, the magnetometer must be isolated from external magnetic field sources. In order to provide a magnetic field-shielded environment, the magnetometer was placed into a six-layered mu-metal chamber, and the voltage output of the relevant axis was connected to the input of the Agilent 35670A dynamic signal analyzer (Agilent Technologies Inc, Santa Clara, CA, USA). Since the voltage noise density (V/√Hz) of the magnetometer can be measured with the signal analyzer, the collected data from the analyzer was divided by the scale factor of each axis to reach the magnetic field noise density (nT/√Hz). The noise spectra of all three axes of the magnetometer are given in Figure 10. The magnetometer noise levels @1Hz were determined to be 1.1 nT/√Hz, 1.3 nT/√Hz, and 2.1 nT/√Hz for the x-, y-, and z-axes, respectively. Duplicate tests have been conducted to ensure the accuracy of the results, and the same results have been found. One of the reasons for the higher noise level observed in the z-axis may be due to the fact that the pick-up coil of this axis is the outermost one (i.e., far from the sensing core). On the other hand, an important issue in noise analysis is the DC magnetic field component in the measurement area. No matter how well we insulate with mu-metal, there may still be magnetic leaks, which can negatively affect the actual noise level of the sensor. While the noise levels of the x- and y-axes are almost the same, another reason for the high noise level seen in the z-axis may be that this axis faces the cover side of the mu-metal chamber. That is, the magnetic leakage along the z-axis may be greater than that along the x- and y-axes. It is worth noting that several methods can reduce the noise level to a few tens of pT, such as annealing the cores between 200 °C and 300 °C to remove structural defects or increasing the ring diameter to reduce stress on the magnetic core. However, these approaches are not the primary focus of the present work.

#### 2.2.4. Bias

To determine the zero-bias field of the magnetometer, it was centered in the Helmholtz coil system shown in Figure 6, with the sensor aligned parallel to the coils. The proper angular alignment of the sensor relative to the coil is of great importance in this measurement. The measurements were repeated five times to check the accuracy. It is also important for this measurement that there should be no magnetic objects in the immediate vicinity of the sensor. The initial voltage readout (V_1_) was recorded by a sensitive digital multimeter (DMM). A current was then applied to the Helmholtz coil to generate a +300 µT field, which can be adjusted higher or lower depending on the requirements. The field was turned off after a short wait. The output voltage values were read again from the DMM and recorded (V_2_). The same process was repeated for a −300 µT field, and the output values were recorded (V_3_). As the last step, ∆V values were calculated by subtracting the V_2_ and V_3_ values from the V_1_ voltage value and converted to magnetic field units by using the scale factor. The maximum bias was determined to be 14.6 nT on the z-axis. We must note here that the bias value within the limits of the world’s magnetic field, <60 µT, remains below the noise levels of the magnetometer (≤2 nT).

#### 2.2.5. Cross-Field Effect

**Figure 11:**
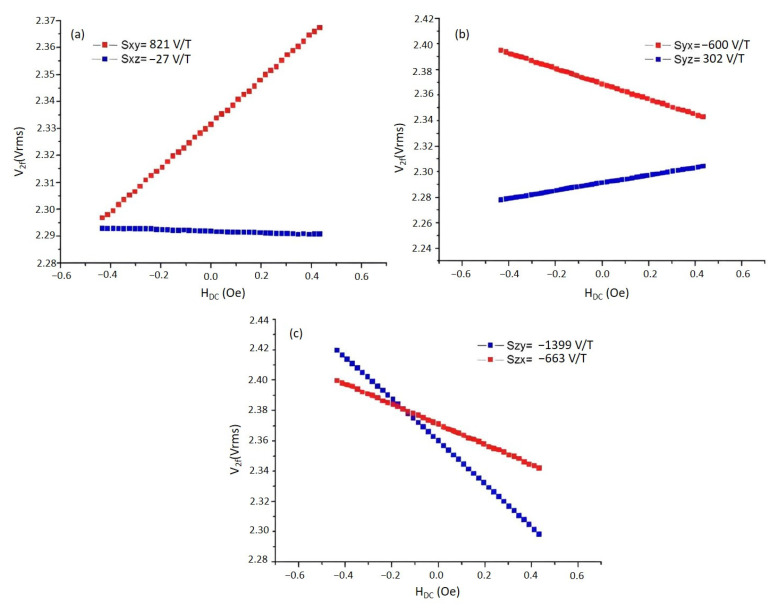
The cross-field effect measurements for (**a**) x-, (**b**) y-, and (**c**) z-axes of the designed magnetometer. Note: 1 Oe = 10^−4^ T.

Determining the cross-field effect, known as the sensitivity of sensors to magnetic fields perpendicular to the sensing direction, is a necessary measurement to keep the total measurement error of the magnetometer at a minimum level. The procedure followed in these measurements is as follows: Before starting the measurement, the magnetometer is placed inside the mu-metal, and the output voltage of the relevant axis is recorded in this zero-field environment. The magnetometer is then placed at the center of the Helmholtz coil system in Figure 6 to determine the cross-field effect. The change in the sensor output signal values is recorded by applying current to the related coil to create a magnetic field perpendicular to the sensor sensing direction. For example, for the sensor whose sensing direction is denoted as x, first, a magnetic field is applied in the y-axis direction, and the output voltage of the x-axis is recorded. The S_xy_ value is obtained from the slope of the V_x_–H_y_ curves. In the same way, a magnetic field is applied along the z-axis, and the S_xz_ value is determined from the slopes of V_x_–H_z_. The important point in cross-field effect measurements is to comply with the right-hand rule. This is repeated for S_yx_, S_yz_, S_zx_, and S_zy_. Figure 11 shows the results of cross-field effect measurements. As seen from Figure 11, scale factors of the cross-field curves may take negative and positive values. For instance, while S_yx_ is −600, V/T it is 302 V/T for S_yz_. It is understood that the cross-fields may affect the magnetometer’s output voltage by up to 4.7%, and their contribution to the error budget can be minimized by putting these values into the matrix given in the model function (see Section 2.2.9).

#### 2.2.6. Temperature Dependency

**Figure 12:**
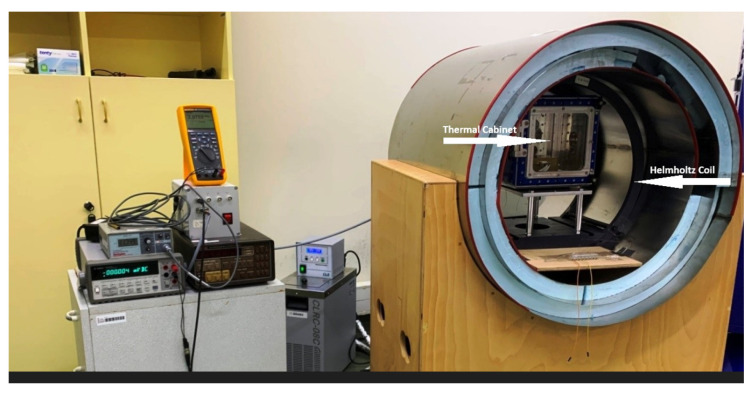
Magnetic-field-shielded thermal vacuum test system.

**Figure 13:**
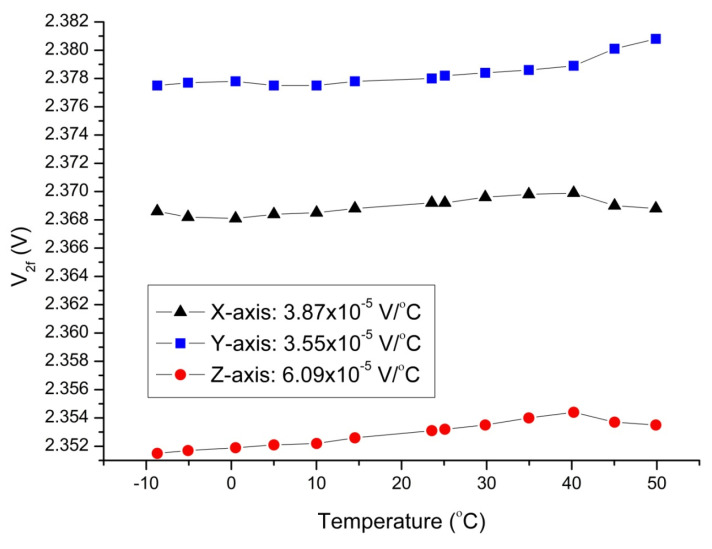
The voltage outputs of the axes of the magnetometer as a function of temperature.

One of the main parameters for the characterization of the magnetometers is its temperature dependency. This parameter is very important for applications exposed to varying temperatures (e.g., space exploration, underground drilling, etc.). For this purpose, a thermal vacuum test system was established in the laboratory, which also provides shielding from the world’s magnetic field (Figure 12). In this system, cabin pressure can be reduced below 10^−5^ mbar. In order to perform tests under a magnetic field, the thermal cabin was placed at the center of a calibrated Helmholtz coil system. The graphs showing the changes in the output voltages of the magnetometer in the temperature range of −10 °C to 50 °C are given in Figure 13. As shown by this measurement, the output voltage of the z-axis changes by a maximum of 4 mV with a 60 °C temperature change, corresponding to approximately 120 nT. The almost linear relationship between the output voltage and the temperature is advantageous in minimizing this error source caused by temperature change. The slopes of the best-fit curves obtained in units of V/°C (inset of Figure 13) for each axis were divided by the scale factor of the relevant axis to obtain the temperature dependency of the sensor in units of magnetic field. It was found to be 1.32 × 10^−3^ μT/°C, 1.23 × 10^−3^ μT/°C, and 2.09 × 10^−3^ μT/°C for the x-, y-, and z-axes, respectively. These values are really quite small, and the resistor of the pick-up circuit has a crucial impact on these values. The greater this resistance, the better the temperature independence can be. This resistance has a crucial impact on the scale factor and sensitivity of the sensor, and hence, it is important for the designer to find the most appropriate value.

#### 2.2.7. Bandwidth

Determining the data reading speed, readout frequency, or bandwidth of a developed magnetometer is another important step in characterization measurements. This indicates how well the magnetometer follows rapid changes in the magnetic field. For this purpose, the measurement axis of the magnetometer is placed in a central position of the Helmholtz coil with parallel alignment to the created AC magnetic field. Then, the output voltage of the magnetometer is zeroed by rotating the Helmholtz coil until a zero-field value is read from the multimeter. When the nulling process of the relevant axis is completed, the magnetometer output is connected to the oscilloscope, and the oscilloscope channel is switched to DC coupling mode. AC current at different frequencies is applied using a calibrated function generator along the relevant axis of the Helmholtz coil. To determine the bandwidth of the magnetometer, the frequency of the AC signal driven to the Helmholtz coil is compared with the frequency of the signal from the magnetometer. If the frequency of the magnetometer output signal is different from the sine form of the signal applied to the coil, then the magnetometer cannot follow the external magnetic field, which determines the measurement speed of the device. This technique, referred to as the sinus-following technique, identifies inconsistencies between the applied signal and the magnetometer output, which indicate the magnetometer’s inability to accurately follow the external magnetic field. It is important to note that if the amplitude of the signal at a certain frequency driven into the Helmholtz coil is not high enough, then the field created may be low. Since the number of windings of the Helmholtz coil is high, the inductive reactance increases with frequency, resulting in insufficient current being driven into the coil. This can prevent accurate detection of the magnetometer’s measurement speed. Increasing the amplitude of the driven current using an amplifier, or decreasing the inductive reactance of the coil with the help of an appropriate capacitance, can solve this problem.

**Figure 14:**
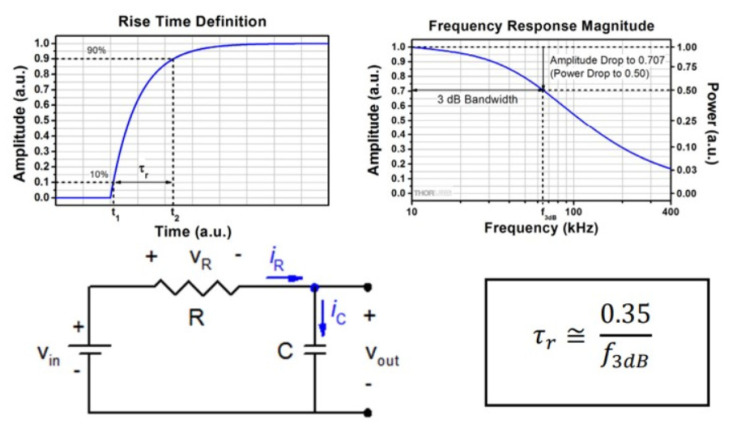
Relationship between bandwidth and rise time.

There is an alternative measurement technique to overcome such inductance problems in the sine-following technique, and it may also be helpful to cross-check the accuracy of the results. In this method, the relationship between the rise time (τ) of the output signal and the bandwidth (BW) is used (Figure 14). Rise time and the 3 dB bandwidth are two closely related parameters used to define the limit of the system’s ability to respond to sudden changes in the input signal. Since it is possible to know only one of these parameters or to find them using available resources, a mathematical expression is used to relate the two. Rise time and the 3 dB bandwidth are inversely proportional, and the proportionality constant is ~0.35 when the response of the system resembles an RC low-pass filter (first-order systems). Rise time is measured by time, while the 3 dB bandwidth is measured by frequency. Rise time is the duration it takes for the output signal to transition from 10% to 90% of its final value in response to an input step function. The 3 dB bandwidth is the frequency at which the system’s response falls to 70.7% of its maximum value. The RC circuit shown in Figure 14 is not part of the actual measurement setup but represents a theoretical equivalent model used to approximate the time-domain and frequency-domain behavior of a first-order low-pass system. This representation is introduced solely to support the analytical derivation of the inverse relationship between rise time and 3 dB bandwidth. The inversely proportional relationship between rise time and 3 dB bandwidth can be derived by considering the time and frequency response of an ideal RC low-pass filter consisting of a resistor and capacitor in series. In this theoretical context, the rise time and the −3 dB bandwidth are not measured directly from a physical RC circuit but inferred using the standard analytical expression valid for first-order systems. The relationship is mathematically expressed as

(3) $$\tau =\frac{0.35}{f_{3\mathrm{dB}}}\;\;\;\;\Rightarrow \;\;\;\;\;{\;f}_{3\mathrm{dB}}=BW\;=\frac{0.35}{\tau }$$

where τ is the rise time between 10% and 90% on the rising edge of the output signal, and f_3dB_ is the 3 dB bandwidth. This relationship is valid for many first-order electrical systems.

In this technique, the magnetometer is initially placed in a central position with the measurement axis parallel to the field created in the Helmholtz coil. Then, current is applied to the coil with a Keithley 220 DC current source, creating a magnetic field high enough to clearly see the magnetometer response. As soon as current is applied to the coil, the signal output of the magnetometer connected to the oscilloscope is recorded by triggering the run–stop button of the oscilloscope. After evaluating the rise time of the captured signal, the bandwidth is calculated using Equation (3). It is crucial in this technique that the reference coil’s time constant is much smaller than that of the magnetometer to ensure accurate measurements. This can be achieved by reducing the inductance (L)-to-resistance (R) ratio in the reference coil circuit. Since the time constant in LR circuits is L/R, it can be reduced by connecting a serial resistor to the reference coil.

**Figure 15:**
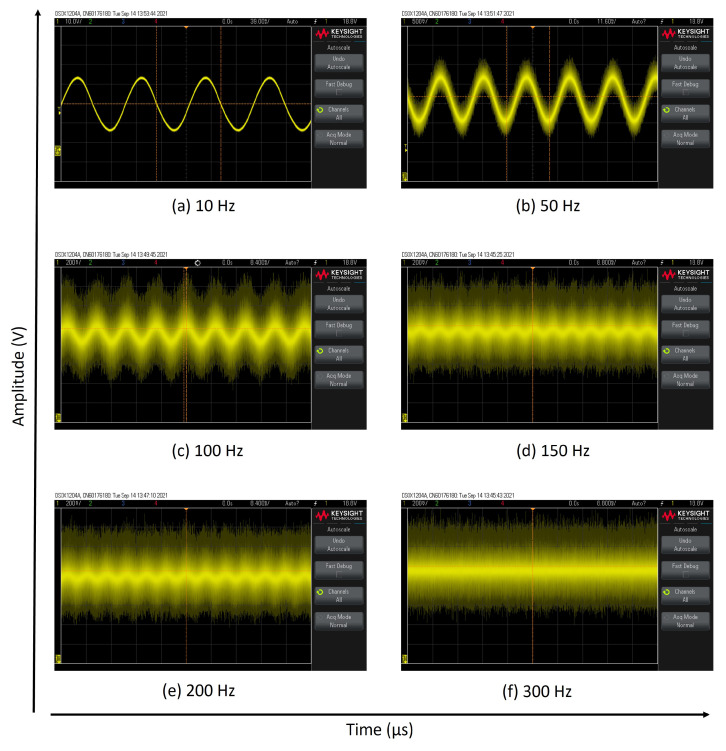
(**a**–**f**) The bandwidth measurements by the sinus-following technique for 10 Hz, 50 Hz, 100 Hz, 150 Hz, 200 Hz, and 300 Hz.

As shown in Figure 15a,b, our measurements with the sinus-following technique reveal that our designed magnetometer can follow the ambient fields of 10 Hz and 50 Hz easily. It can actually monitor fields up to 200 Hz, although it is noisy (Figure 15a–f). If a second channel was used for triggering, then the sinus-following test could be investigated with a better outcome by employing signal averaging. As mentioned above, its accuracy is limited by the inductance of the coil system and may be improved by using reference coils having much lower inductances.

**Figure 16:**
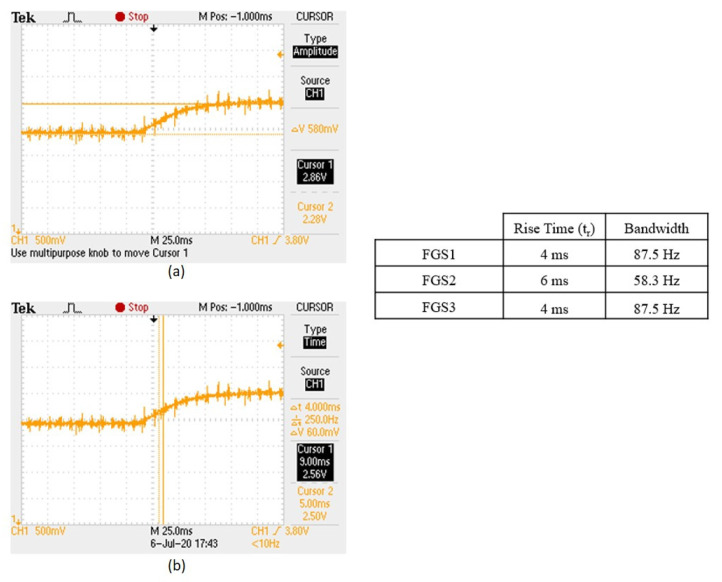
The bandwidth measurements by the rise time technique: (**a**) upper–lower voltage limits; (**b**) rise time.

As shown in Figure 16a,b, our measurements using the rise time method reveal that the designed magnetometer can follow ambient fields at different frequencies. The rise time measurements indicate that the magnetometer can effectively monitor fields up to 87.5 Hz with a rise time of 4 ms for FGS1 (commercially available cobalt-based alloy containing CoFeSiNiB) and FGS3 (FINEMET-type Fe_71.5_Si_15.5_B_9_NbCu_3_ iron-based alloy also produced by the Slovak Academy of Sciences), and up to 58.3 Hz with a rise time of 6 ms for FGS2 (rich amorphous ribbon with Co_69_Fe_2_Cr_7_Si_8_B_14_ composition, developed by the Slovak Academy of Science). This demonstrates that the magnetometer provides consistent performance for moderate frequency detection. These results confirm that similar to the sinus-following technique, the rise time method shows the magnetometer’s capability to track magnetic fields at lower frequencies accurately, providing a clear indication of its response speed and limitations at higher frequencies. To summarize the two measurement methods suggested above, the sinus-following technique tests the magnetometer’s ability to track continuous, sinusoidal changes in the magnetic field across a range of frequencies. It directly assesses the magnetometer’s capability to handle real-world dynamic scenarios where magnetic fields may vary sinusoidally, such as in electronic and industrial environments. However, this method can be influenced by the properties of the coil system (e.g., inductance and resistance), potentially affecting the accuracy of the measurements if not properly controlled. On the other hand, the rise time technique evaluates the speed of the magnetometer’s response to abrupt changes in magnetic field strength, providing a measure of the system’s overall immunity and frequency bandwidth. This method is useful for assessing the quickness and sharpness of the sensor’s response, which is critical in applications requiring fast detection of magnetic field changes. However, this assumption relies on the system behaving like a first-order low-pass filter, which may not hold true for complex or nonlinear systems and might not fully capture the sensor’s performance under continuously varying magnetic fields. Combining both methods offers a comprehensive assessment of the magnetometer’s dynamic performance, ensuring robustness and reliability for various applications.

Determining the best-suited measurement method for the described fluxgate magnetometer depends on the specific applications and performance requirements of the magnetometer. If the magnetometer is intended for applications involving rapidly changing or fluctuating magnetic fields where it needs to accurately track changes without phase shift or lag, then the sinus-following method might be more appropriate. This method provides a direct assessment of how well the magnetometer can keep up with sinusoidal variations in the magnetic field, which is typical in many practical applications like navigation and tracking. However, if the primary requirement is for the magnetometer to respond quickly to sudden changes in the magnetic field, such as detecting transient magnetic anomalies or quick disturbances, then the rise time to 3 dB bandwidth method would be more suitable. This method will give a clear indication of how quickly the sensor can react to sudden changes, an essential factor in high-speed detection scenarios. For the proposed fluxgate magnetometer, where detailed calibration and characterization are emphasized, incorporating both methods could provide a comprehensive view of its dynamic capabilities. Using both methods would allow for a thorough assessment of the magnetometer’s performance across a range of operational conditions, ensuring robustness and reliability in its specified application field.

#### 2.2.8. Power Consumption, In-Rush Current, and Supply Voltage Dependency

The last three characterization measurements for a developed magnetometer (also applicable to most electronic devices) are the determination of power consumption, in-rush current measurement, and the extent to which the device is affected by fluctuations in the supply voltage. In order to determine the power consumption, the data and power lines of the magnetometer were separated, and the circuit supply was provided by a dual-output adjustable power supply. While the negative supply of the power line is connected directly to the power supply, the positive supply of the magnetometer is connected serially to the power supply via DMM in order to read the current drawn by the magnetometer. By applying ±12 V from the power supply, the current value read from the DMM was recorded, and the magnetometer power consumption was calculated with the equation P = V × I. The current drawn from the power supply of the developed magnetometer was measured to be 33.80 mA, and the power consumption was determined to be ~0.4 W. It must be noticed that the value of the resistance connected to the entrance of the excitation tank is decisive for the power consumption. One can decrease the power consumption more by increasing the resistance. However, in this case, the sensitivity of the sensor may become worse. So, a balance needs to be achieved.

**Figure 17:**
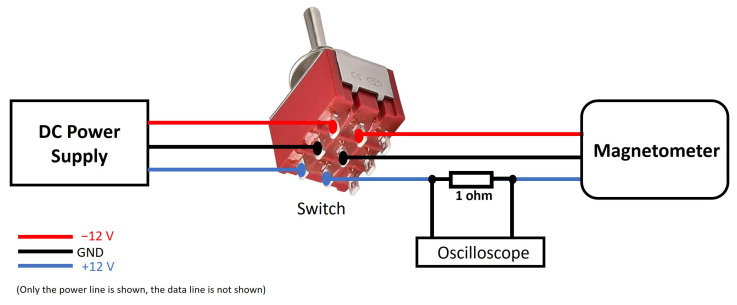
In-rush current measurement setup on the positive power line.

**Figure 18:**
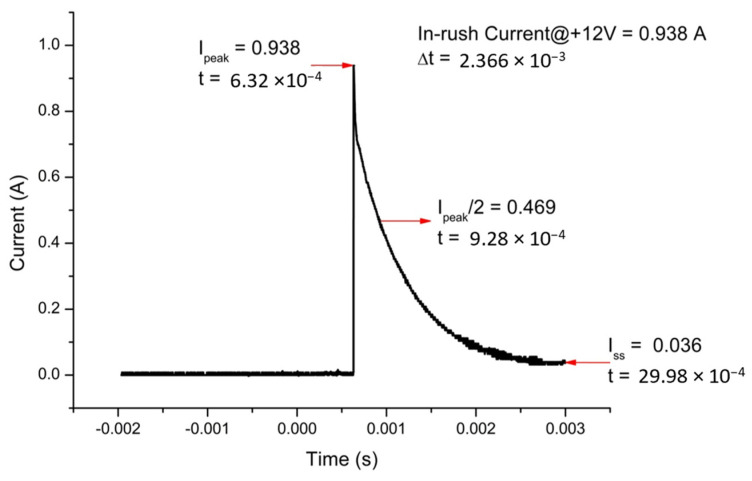
In-rush current on the positive power line of the designed magnetometer.

In-rush current is the maximum instantaneous input current drawn by the magnetometer when it is first turned on. It must be measured and, if necessary, should be taken under control. They generally have much higher in-rush currents than steady-state currents due to the charging current of the input capacitance when first energized. Most of this in-rush current is due to the input capacitor placed on the supply line. Introducing a high resistance between the input power and the capacitor can increase the resistance of power-on, resulting in a decrease in in-rush current. Using an in-rush current limiter for this purpose can be helpful as it can provide the initial resistance required. The setup shown in Figure 17 can be used for in-rush current measurement. In this setup, the 1-ohm resistor limits the in-rush current when the switch is activated and power is applied to the magnetometer. This prevents sudden current surges that could potentially damage sensitive components or distort the measurement. Secondly, this resistor, in combination with the inductive components of the magnetometer, forms an RL circuit, which affects the time constant (τ = L/R) of the system. As such, it influences the rise time and decay behavior of the impulse response observed on the oscilloscope. For this purpose, a 1-ohm resistor and an oscilloscope parallel to the resistance are connected to the positive power line between the switch and the magnetometer. After the connections are completed, the switch is kept OFF and the DC power supply is kept ON. As soon as the switch is opened, the image is captured by pressing the run/stop button of the oscilloscope and the value at the point where the signal peaks is calculated. The oscilloscope image of the magnetometer, which consumes approximately 35 mA from the positive power line during normal operation but draws 0.93 A in-rush current, is given in Figure 18. The in-rush current of the negative power line can also be measured in a similar manner. We found it to be 0.88 A. It must be noted that in-rush current times (∆t) on the positive and negative power lines are calculated between the initial current drawn by the device (I_peak_) and the time during which it drops to half of this value (I_peak_/2).

**Figure 19:**
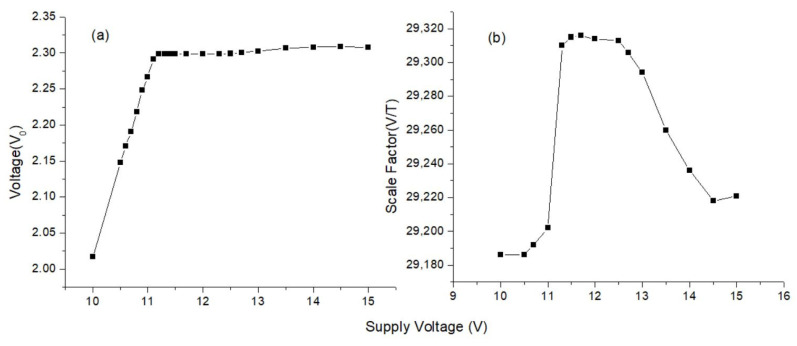
(**a**) Zero-field voltage value. (**b**) Scale factor graph for the x-axis at different supply voltage values of the magnetometer.

A mechanism similar to that in power measurement was established to examine the effect of fluctuations in the supply voltage on the accuracy of the magnetometer. As in power measurement, the data and power lines of the magnetometer were separated from each other. The negative and positive feeds of the power line were connected to the power supply, and the data line was connected to the DMM for reading. Then, the magnetometer was placed in the mu-metal, the normal operating voltage of the magnetometer, ±12 V, was initially supplied from the power supply, and the axis voltage output values in the zero-field were recorded. Afterwards, the same process was repeated at different supply voltage values. In order to examine the measurement performance of the magnetometer depending on the supply voltage, scale factor measurement was conducted at these voltage values. Measurements were made between ±15 V and ±10 V supply voltages, and the results of x-axis are given in Figure 19. It was observed that there was no serious loss in magnetometer performance when the supply voltage value fluctuated between ±11 V and ±13 V. Our experimental results indicate that the capacitances in the tanks of the excitation and pick-up circuits and the resistance in the tank of the pick-up circuit play critical roles in the robustness against power fluctuations. However, in this case too, the sensitivity may become worse and needs to be optimized.

#### 2.2.9. Model Function

A model function is needed to establish a relationship between the magnetometer’s output voltage and the measured magnetic field. However, initially, the inverse of the matrix below must be found since the elements of the matrix, determined from the previous experiments, have units of V/T.

(4) $$\left[\begin{array}{ccc} S_{X} & S_{XY} & S_{XZ}\\ S_{YX} & S_{Y} & S_{YZ}\\ S_{ZX} & S_{ZY} & S_{Z} \end{array}\right]$$

After filling the matrix above, it becomes

(5) $$\left[\begin{array}{ccc} 29402 & 821 & -27\\ -600 & 28871 & 302\\ -663 & -1399 & 29307 \end{array}\right]\;V/T$$

The inverse of this matrix is

(6) $$\left[\begin{array}{ccc} 33.99 & -0.964 & 0.041\\ 0.698 & 34.59 & -0.356\\ 0.802 & 1.629 & 34.10 \end{array}\right]\mu T/V$$

The model functions for each axis can now be found from the matrix below

(7) $$\left[\begin{array}{c} H_{X}\\ H_{Y}\\ H_{Z} \end{array}\right]=\left[\begin{array}{ccc} {S^{\prime }}_{X} & {S^{\prime }}_{XY} & {S^{\prime }}_{XZ}\\ {S^{\prime }}_{YX} & {S^{\prime }}_{Y} & {S^{\prime }}_{YZ}\\ {S^{\prime }}_{ZX} & {S^{\prime }}_{ZY} & {S^{\prime }}_{Z} \end{array}\right]\left[\begin{array}{c} U_{X}-U_{X0}\\ U_{Y}-U_{Y0}\\ U_{Z}-U_{Z0} \end{array}\right]$$

where, for instance, S′_x_, S′_y_, and S′_z_ are scale factors of the axes in units of μT/V; S′_XY_, S′_XZ_, S′_YX_, S′_YZ_, S′_ZX_, and S′_ZY_ are the cross-field effects in units of μT/V; U_X_, U_Y_, and U_Z_ are voltage values read from the axis outputs during measurement in units of V; U_X0_, U_Y0_, and U_Z0_ are zero-field values of the axis outputs in units of V. After inputting the numerical values of the matrix elements, we obtain

(8) $$\left[\begin{array}{c} H_{X}\\ H_{Y}\\ H_{Z} \end{array}\right]=\left[\begin{array}{ccc} 33.99 & -0.964 & 0.041\\ 0.698 & 34.59 & -0.356\\ 0.802 & 1.629 & 34.10 \end{array}\right]\left[\begin{array}{c} U_{X}-2.3759\\ U_{Y}-2.3693\\ U_{Z}-2.3574 \end{array}\right]$$

Then, first order equations are obtained:

H_x_ = 33.99∙U_x_ − 0.964∙U_y_ + 0.041∙U_z_ − 78.569 (9)

H_y_ = 0.698∙U_x_ + 34.59∙U_y_ − 0.356∙U_z_ − 83.021 (10)

H_z_ = 0.802∙U_x_ + 1.629∙U_y_ + 34.10∙U_z_ − 86.152 (11)

In these equations, H values are in units of μT. These measurements were carried out at temperatures of 25.2 °C. So, we need to add the contribution of the operation temperature to these equations. The equations above were modified with the contribution of temperature as

H_x_ = 33.99∙U_x_ − 0.964∙U_y_ + 0.041∙U_z_ − 78.569 + (T − 25.2) (1.32 × 10^−3^ μT/°C) (12)

H_y_ = 0.698∙U_x_ + 34.59∙U_y_ − 0.356∙U_z_ − 83.021 + (T − 25.2) (1.23 × 10^−3^ μT/°C) (13)

H_z_ = 0.802∙U_x_ + 1.629∙U_y_ + 34.10∙U_z_ − 86.152 + (T − 25.2) (2.09 × 10^−3^ μT/°C) (14)

With these equations, the error coming from the operation temperature can be decreased from 120 nT to less than 5 nT for the z-axis at a temperature range of −10 °C to 50 °C.

## 3. Conclusions

In this paper, the design features of the three-axis fluxgate magnetometer have been described with its main parameters, as well as the results of tests of the manufactured sample of the magnetometer. In particular, data on the linearity and resolution of the sensor, the noise level, the zero-level instability and temperature dependence of the readings, the influence of power supply instability, and the power consumption by the sensor are given. On the basis of the obtained results, analytical expressions are offered, which allow for the consideration of the influence of external factors and increase the accuracy of the determination of all three components of the vector of the measured magnetic induction. This study has especially highlighted several critical factors that significantly influence the performance and reliability of the sensor. Among these, adjusting the resistance and capacitance values in the excitation and detection coils is crucial for optimizing the sensor’s functionality to meet specific application needs. The resistance and capacitance within these coils play pivotal roles, directly impacting the magnetometer’s ability to remain stable under temperature and power fluctuations. Proper tuning of these components not only ensures the independence of the magnetometer from external disturbances but also aids in fine-tuning the power consumption and the overall sensitivity of the device. The present magnetometer has been working reproducibly for more than 6 months after making the proper tuning of the resistances and capacitances. These adjustments are essential for minimizing the noise level, which is a significant determinant of the sensor’s accuracy and reliability. Depending on the application area, whether it be space exploration, underground resource detection, or biomedical applications, the dominance of certain sensor characteristics such as sensitivity, range, and noise immunity needs to be considered. Each application demands specific resistance and capacitance settings to balance sensitivity with noise levels and operational stability. Therefore, when designing and calibrating fluxgate magnetometers, researchers must carefully consider how these elements, resistance and capacitance, align with the operational demands of their intended application. By doing so, they can ensure that the magnetometer not only performs optimally under laboratory conditions but also maintains its performance integrity in the field, providing reliable and precise measurements crucial for the success of the application. Additionally, this study provides a detailed overview of the critical processes involved in the characterization of three-axis magnetometers for low-magnetic-field measurement. So far, such an in-depth article has been lacking in the scientific literature on fluxgate magnetometers. As a result, this paper will be very useful for the relevant people, as it reveals the fundamental problems encountered in three-axis very low-magnetic-field measurements and presents the critical components in the design of these sensors.

## Data Availability

Data are contained within the article.

## Abbreviations

The following abbreviations are used in this manuscript:

PCB	Printed Circuit Board
SMD	Surface Mount Device
FGS	Fluxgate Sensor
